# Spatiotemporal thermal variation drives diversity trends in experimental landscapes

**DOI:** 10.1111/1365-2656.13867

**Published:** 2022-12-15

**Authors:** Ellie Wolfe, Francesco Cerini, Marc Besson, Duncan O'Brien, Christopher F. Clements

**Affiliations:** ^1^ School of Biological Sciences University of Bristol Bristol UK; ^2^ Sorbonne Université CNRS UMR Biologie des organismes marins, BIOM Banyuls‐sur‐Mer France

**Keywords:** community, corridor length, dispersal, protist microcosm, temperature variation

## Abstract

1. Temperature is a fundamental driver of species' vital rates and thus coexistence, extinctions and community composition. While temperature is neither static in space nor in time, little work has incorporated spatiotemporal dynamics into community‐level investigations of thermal variation.
2. We conducted a microcosm experiment using ciliate protozoa to test the effects of temperatures fluctuating synchronously or asynchronously on communities in two‐patch landscapes connected by short or long corridors.
3. We monitored the abundance of each species for 4 weeks—equivalent to ~28 generations—measuring the effects of four temperature regimes and two corridor lengths on community diversity and composition.
4. While corridor length significantly altered the trajectory of diversity change in the communities, this did not result in different community structures at the end of the experiment. The type of thermal variation significantly affected both the temporal dynamics of diversity change and final community composition, with synchronous fluctuation causing deterministic extinctions that were consistent across replicates and spatial variation causing the greatest diversity declines.
5. Our results suggest that the presence and type of thermal variation can play an important role in structuring ecological communities, especially when it interacts with dispersal between habitat patches.

## Graphical abstract

Temperature variability is increasing due to global climate change, but limited work has investigated the effects at the metacommunity level. This study uses a multiple trophic level experimental system to demonstrate that the type of thermal variation—spatial, temporal, or both—influences diversity trends and community composition through time.

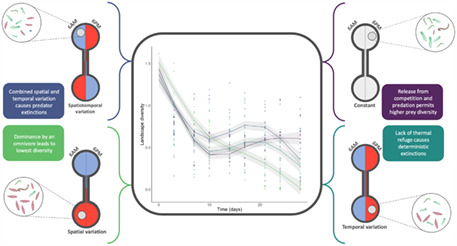

## 1 INTRODUCTION

The role of variable environmental conditions in shaping species diversity within ecosystems is a prominent theme in ecology and conservation biology (Bernhardt et al., 2020; Trew & Maclean, 2021). Such topics have been investigated with macro‐ (Fried‐Petersen et al., 2020) and micro‐scale (Leary et al., 2012) approaches, and tackled at the population (Gonzalez & Holt, 2002) and community level (Shurin et al., 2010). To date, the idiosyncrasy of how fluctuating conditions affect the coexistence of species in communities has resulted in mixed findings (Adler & Drake, 2008; Chesson & Huntly, 1997; Connell, 1978). Environmental variation has been shown to lower species fitness and diversity due to differential responses to new conditions and increased risk of stochastic extinctions (Adler & Drake, 2008; Rasconi et al., 2015). On the other hand, it may also facilitate temporal niche partitioning among species that perform best in different conditions, thus avoiding competitive processes and promoting diversity (the storage effect; Chesson & Warner, 1981; Snyder & Adler, 2011). This role of environmental variability as a facilitator of coexistence or a promoter of extinctions illustrates that insights on this topic are needed in the face of current and future global change scenarios where variability of environmental factors such as climate and habitat availability is expected to increase (Bathiany et al., 2018).

Among the environmental conditions which shape the abundance and distribution of organisms, temperature is understood to be of fundamental importance. The direct effects of temperature in determining the growth rates and metabolic activity of individuals (Brown et al., 2004) scale up to the community level, with such changes impacting the structure of communities through changes not only in species survivorship (Trew & Maclean, 2021) but also in the strength of interactions between species (Meester et al., 2015). Consequently, when temperature is variable, it can greatly alter the dynamics of communities (at the local scale) and metacommunities (at the landscape scale). Locally, temporal temperature variation can promote coexistence between competing species if they respond asynchronously to the temperature variation (Yachi & Loreau, 1999). This was demonstrated in lake zooplankton, where temperature variation increased diversity by facilitating niche partitioning (Shurin et al., 2010). Conversely, temperature variation can have a range of detrimental consequences – causing population declines and ultimately local extinctions by reducing species' ability to withstand diseases (e.g. in amphibians, Rohr & Raffel, 2010), causing community destabilisation by favouring fast‐growing species that dominate a community (e.g. in phytoplankton, Rasconi et al., 2017), and decreasing species richness by reducing reproduction (e.g. in grasslands, Zhang et al., 2018). Modelling has revealed that these processes can occur in tandem, with environmental variation simultaneously increasing risk of stochastic extinction and permitting coexistence (Adler & Drake, 2008). This highlights the importance of tracking the responses of individual species to temperature variation through time in order to understand what drives community‐level responses.

However, to date, experimental studies investigating the effects of temperature variation at the community level tend to do so in simple single‐patch systems (Leary & Petchey, 2009; Rasconi et al., 2017), overlooking the important role of spatial dynamics in driving patterns of response. This oversight is particularly pertinent given that, in natural systems, landscapes are thermally heterogeneous, with temperature being a key driver of species distributions (Hutchins, 1947; Parmesan & Yohe, 2003). Individuals track favourable conditions to occupy a suitable thermal niche (Chesson & Warner, 1981; Leibold et al., 2004), and so temperature variation can be expected to alter the spatial dynamics of species at the landscape scale. For example, individuals may move towards an area of favourable temperature where growth rates will increase (Barton & Ives, 2014) or seek refuge to escape predation or competition which may have increased due to temperature change (Daugaard et al., 2019; Jiang & Kulczycki, 2004; Walberg & Green, 2021). This movement towards favourable‐temperature areas can simply reduce the occupiable niche space for interacting species, exacerbating the effects of an interaction (Salt et al., 2017). Conversely, landscape‐level processes such as these can promote the overall stability or diversity of a system subjected to environmental variation. For instance, if temperature change causes localised extinctions, rescue effects can occur whereby colonisation from non‐affected patches may ensure persistence across the landscape (Brown & Kodric‐Brown, 1977). The importance of studying the interaction between dispersal and environmental variation is illustrated by an experiment revealing that temporally autocorrelated thermal variation can enhance the beneficial effects of immigration in sustaining an inferior competitor which would otherwise be outcompeted (Long et al., 2007). The importance of these spatial processes in maintaining diversity in the face of temperature variation illustrates the requirement for further investigations into these effects on communities in multi‐patch landscapes.

Simplified experimental approaches such as microcosms and mesocosms are a valuable tool in this sense (Arnott et al., 2021) because they permit the creation of artificial ecosystems with customised landscape structures (Wolfe, Hammill, et al., 2022), manipulation of communities according to species traits, abundance and assembly order (Clements et al., 2013), and precise modification of temperature (Jiang & Morin, 2007). These approaches have been invaluable in their ability to describe the effects of temperature on fine‐scale processes such as coexistence of competing species (Jiang & Morin, 2007), predator–prey dynamics (Salt et al., 2017) and changes in body size (Laakso et al., 2003).

Here we employ this classic and highly flexible system to explore the effects of temperature variability on biodiversity using multi‐species freshwater ciliate experimental metacommunities. The temperature variation treatments encompassed three realistic scenarios experienced by natural communities – spatial (‘static difference’), synchronous (‘fluctuating synchronous’) and asynchronous (‘fluctuating asynchronous’). We provide additional complexity by 3D printing custom‐designed landscapes of patches connected by corridors of varying lengths, enabling us to investigate the role of dispersal‐driven spatial dynamics. By monitoring the abundance of each species at regular intervals throughout the duration of the 4‐week experiment, we were able to investigate species dynamics over approximately 28 generations (Clements et al., 2013) and measured these effects on patch—and landscape—level diversity. In addition to monitoring between‐treatment differences, we also investigated the treatments' effects on within‐treatment variation, enabling us to consider whether the effects were deterministic, causing consistent changes across replicates, or stochastic, causing variable changes across replicates. Given the multitude of documented direct (Warren et al., 2006) and indirect (Daugaard et al., 2019) responses of community diversity to temperature, and the non‐linear effect that temperature variation can have on metabolic rates in ectotherms (Martin & Huey, 2008)—we expect all three types of thermal variation to result in higher landscape diversity compared with the control (hypothesis 1). More specifically, the static difference treatment would promote diversity via niche partitioning caused by spatial thermal variation (hypothesis 1a), the fluctuating synchronous treatment would promote diversity through the storage effect caused by temporal thermal variation (hypothesis 1b), whereas the fluctuating asynchronous treatment combines the influence of both niche partitioning and the storage effect caused by spatiotemporal variation (hypothesis 1c). We also predicted that there would be an interaction between temperature treatment and corridor length, where communities in short‐corridor landscapes would be better able to respond to spatial temperature variation in the static difference and fluctuating asynchronous treatments, due to more frequent dispersal, thus increasing diversity (hypothesis 2). Finally, we anticipate each temperature treatment to differ in their influence on biodiversity, but exact hypotheses were not made given the complex responses to temperature variability in multi‐species networks (Petchey et al., 2010). We therefore interpreted the results in a pattern‐to‐process approach.

We consequently provide experimental evidence that the type of thermal variation plays an important role in driving diversity trends, ultimately altering community composition. Our results also question the potential for dispersal to mitigate these effects. Disentangling these responses to different types of thermal variation will bring us one step closer to being able to predict community responses to environmental variation.

## 2 MATERIALS AND METHODS

### 2.1 Experimental design

To investigate the effects of temperature fluctuations and dispersal distance on community diversity we factorially manipulated corridor length and the spatiotemporal variance of temperature in two‐patch experimental landscapes containing freshwater ciliate protozoa. The experimental landscapes, which consisted of two circular patches (diameter 5 cm, depth 2 cm) connected by a linear corridor, were custom‐designed using FreeCAD 3D‐design software (https://www.freecad.org/) and 3D‐printed (Lulzbot TAZ 6) in black PLA filament. The corridor was either 5 cm (‘short’) or 10 cm (‘long’). This difference in corridor length, previously used in experiments of spatial dynamics of the ciliate *Colpidium striatum* (Donahue et al., 2003), is sufficient to induce changes in dispersal rates in some of the species within our community (Table S1; Figure S1). Therefore, in line with research demonstrating that doubling corridor length from 5 to 10 cm hampers habitat choice in another species of ciliate protozoa—*Tetrahymena pyriformis*—thus posing a greater cost to dispersing individuals (Laurent et al., 2020), we chose these lengths to simulate different times taken for the protozoans to move between the two patches. We opted not to include a no‐dispersal control because we wanted to investigate the landscape‐scale effects of thermal variation, which by definition include dispersal between habitat patches (Leibold et al., 2004). We printed 40 landscapes that were divided among two corridor treatments (20 short and 20 long) and four temperature treatments (see below), giving a total of five replicates per corridor: temperature combination.

**FIGURE 1:**
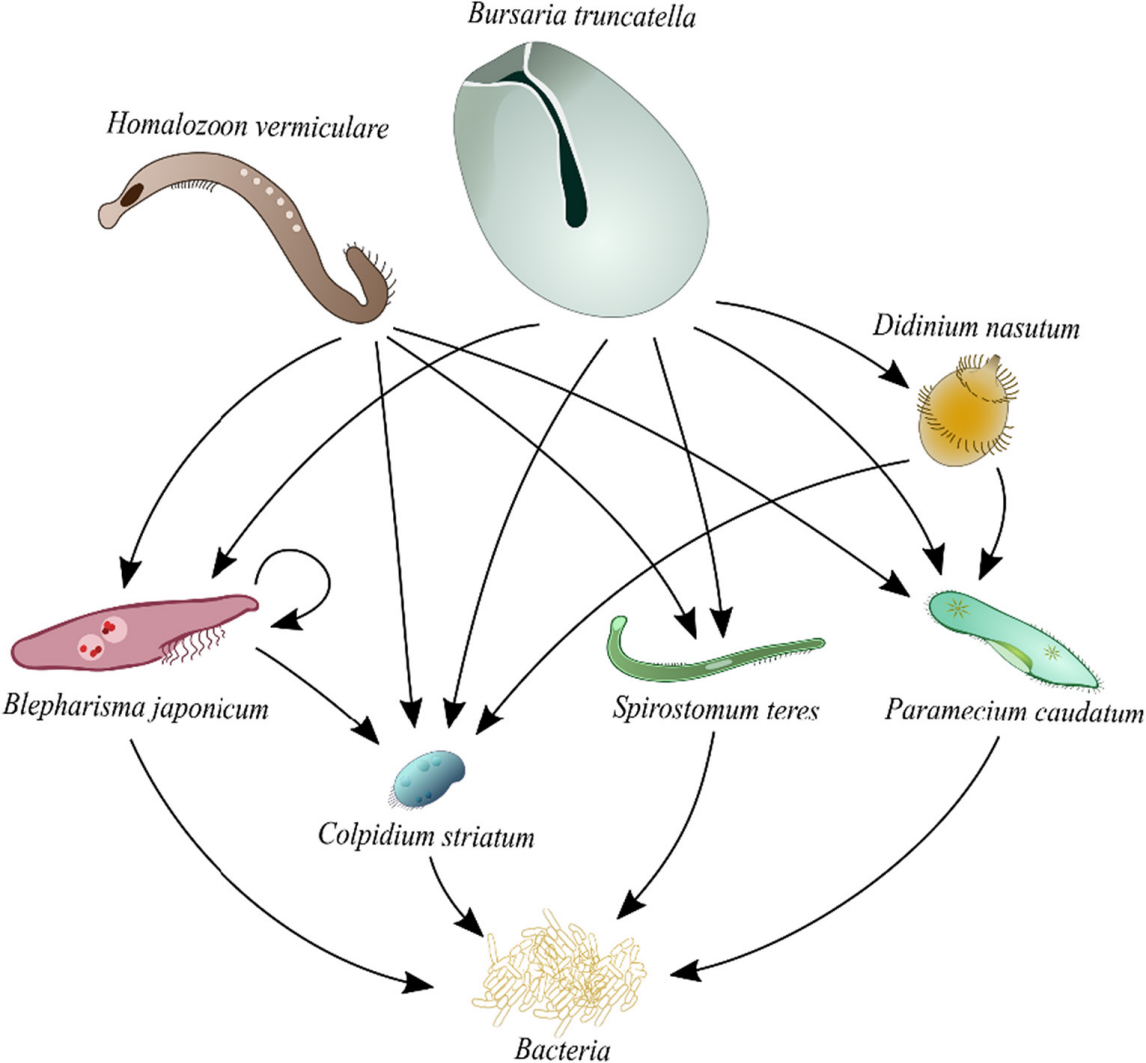
Proposed food web of the seven‐species protist community. Feeding links obtained from Baumberg and Hausmann (2007), Beers (1948), Burkey (1997), Warren et al. (2006) and Worsfold et al. (2009).

In each of these experimental landscapes, we constructed a community consisting of seven species of freshwater ciliate protozoa: two apex predators, one intermediate predator, one omnivore, and three bacterivores (Figure 1) (obtained from Sciento). These species live within a freshwater media made up of crushed protozoa pellets (Blades Biological) dissolved in Chalkley's solution at a concentration of 0.3 g L^−1^ (Clements et al., 2013). This media provides a carbon source on which the two species of bacteria which form the lowest trophic level (*Bacillus subtilis* and *Pseudomonas fluorescens*) can thrive. The media was inoculated with bacteria 3 days prior to the start of the experiment, so that the bacteria levels were sufficient for the protists to survive. On day 0, the start of the experiment, the protist community was added to the experimental landscapes at densities of 1 individual ml^−1^ for the predators and 10 individuals ml^−1^ for the bacterivores and omnivore (Figure 1). To ensure species were randomly distributed across experimental landscapes, we added volumes containing the appropriate number of each species to a sterile flask from high density stock cultures and mixed this thoroughly before distributing evenly across both patches in each landscape. Landscapes were then topped up with the bacteria‐inoculated nutrient media so each landscape contained 40 ml. This meant that the long‐corridor landscapes had a slightly lower media depth (~0.024 cm lower) due to their corridor length, although this effect is negligible. This landscape design meant that the corridors contained the same media as the patches and so were hospitable to dispersing individuals. We deemed this a suitable representation of landscapes because corridors are often designed to provide a safer route for dispersing animals, for example in the form of overpasses and underpasses which enable individuals to cross roads (Simpson et al., 2016).

The 40 experimental landscapes were then equally divided among the four temperature treatments. We opted not to acclimate the stocks prior to experiment set‐up because the average temperature of each landscape over a 24‐h period was 20°C, the same temperature that the stocks were kept at. Additionally, we chose to control for average patch temperature at the landscape level because, as recent evidence has shown (Hammill & Dart, 2022), mean temperature interacts with temperature variation. Our four temperature regimes manipulated either spatial variation in temperature, temporal variation in temperature, both spatial and temporal variation, or neither (acting as a control). The ‘static difference’ treatment manipulated spatial variation in temperature, with one patch in the landscape at 15°C for the entire experimental period and the other at 25°C. The ‘fluctuating synchronous’ treatment manipulated temporal variation in temperature, with both patches varying synchronously between 15 and 25°C every 12 h (changing the temperature at 6 am and 6 pm) for the duration of the experiment. The ‘fluctuating asynchronous’ treatment manipulated both the temporal and spatial variation in temperature simultaneously, with one patch in each landscape at 15°C and the other at 25°C for 12 h each day, and then the temperatures swapped, with temperatures also changed at 6 am and 6 pm. Finally, for the ‘constant’ treatment, we maintained both patches at 20°C—the average microcosm temperature for each treatment outlined above. The constant microcosms were kept in an incubator set to 20°C, while the microcosms subjected to temperature variation were kept in a temperature‐control room set to 15°C, with patches heated to 25°C where necessary using heat mats (AIICIOO) placed under patches controlled by timers. The risk of potential bias arising from such difference environmental conditions (i.e. incubator vs. temperature control room) was minimised by keeping lids on all microcosms at all times, except during sampling. This, in addition to avoid excessive evaporation, reduced the risk of any differences in lighting or airflow. These temperatures were selected to be sufficient to drive differences in growth rates and interaction strengths, but not to directly cause mortality (Table S2; Jiang & Kulczycki, 2004; Leary & Petchey, 2009).

Given the potential role for immigration via dispersal to sustain populations subjected to thermal variation (Long et al., 2007), we factorially crossed the two corridor lengths with the four temperature treatments. The aim of this was to implement an energetic cost associated with moving between different temperatures, imposing a trade‐off to those species that may be positively or negatively affected by temperature. At the start of the experiment one microcosm leaked (static difference short corridor replicate 4), and so was discarded.

The abundances of all protist species were estimated in each patch from a sample of the community twice a week, on Mondays and Thursdays. Prior to sampling we recorded the temperature of each patch and topped the microcosms up with autoclaved distilled water to account for any evaporation. To sample, we mixed the contents of the patch by pipetting up and down, then pipetted a 2 ml subsample on to a sterile petri dish. We then counted abundances of each species using a stereomicroscope. If the abundance of any one species was too high to count (>50 individuals), we took a further subsample of appropriate volume. If the abundance of any one species was too low to ensure it was a true representation of the patch‐level densities (<5 individuals), we counted the number of individuals in the whole patch. Once a week, on Mondays, we replenished nutrients by discarding the 2 ml subsample and replacing this with 2 ml fresh sterile media (autoclaved Chalkley's solution containing 0.3 g L^−1^ crushed protozoa pellets). On Thursdays the subsample was pipetted back into the patch. The experiment lasted for 28 days which equates to approximately 28 protist generations (Clements et al., 2013). Our study did not require ethical approval.

### 2.2 Statistical analysis

Data consisted of abundance time series for each of the seven species within each patch (Figure S2). Shannon diversity (hereafter ‘landscape diversity’; Spellerberg & Fedor, 2003) was estimated using the ‘vegan’ package (Oksanen et al., 2020), resulting in abundance‐weighted species diversity measures for each landscape (pooling the local diversity data of both patches) at all time points. We estimated inter‐patch dissimilarity as the Bray–Curtis dissimilarity between the two patches in each landscape (hereafter ‘beta diversity’), using the ‘betapart’ package (Baselga, 2017). All statistical analyses were conducted using r version 4.1.2 (R Core Team, 2022).

We also used generalised mixed‐effects models (GLMMs) using the ‘glmmTMB’ package (Brooks et al., 2017) to assess the trends in the landscape diversity through time, and how such trends differed across corridor lengths and temperature regimes. The model was initially parameterised with the following structure:

$$\begin{array}{c} \mathrm{Sh}\_\mathrm{div}∼f\left(\text{NumDays},3\right)+\text{Temp}\_\text{Regime}+\text{Corridor}+f\left(\text{NumDays},3\right)\\ :\text{Temp}\_\text{Regime}+f\left(\text{NumDays},3\right):\text{Corridor}+\text{Temp}\_\text{regime}\\ :\text{Corridor}+\text{Temp}\_\text{Regime}:\text{Corridor}:f\left(\text{NumDays},3\right)\\ +\text{ar1}\left(\text{NumDays}\right)+\left(1|\text{Replicate}\right) \end{array}$$

and fit using a Gaussian distribution. ‘*Sh_div*’ indicates the landscape‐level Shannon diversity index response variable, ‘*f(NumDays,3)*’ is a natural cubic spline function with three knots for the time variable ‘*NumDays*’ with days as units (continuous variable), ‘*Temp_Regime*’ is the temperature treatment used in the experiment (categorical variable with four levels) and ‘*Corridor*’ represents whether a long or short corridor separated the two patches (categorical variable with two levels). Interactions between variables are indicated by a colon and represent changes in temporal trend for each categorical level. Replicate was included as a random factor to account for potential non‐independence within replicates. Additionally, a first‐order auto‐regressive covariance structure (ar1(NumDays)) was included to control for serial autocorrelation in the model residuals (Zuur et al., 2009). The use of a spline represents curvilinear trends in response variables better than a linear predictor (Aubry et al., 2009; Hess, 1994) and accounts for autocorrelation between temporally adjacent data points (Simpson, 2018).

We then used a top‐down approach to arrive at the best descriptive models using the corrected Akaike information criterion (AICc) which better performs with small sample sizes (Song et al., 2017). Comparisons of AICc values were used to assess the loss of explanatory power following the removal of a three‐way or two‐way interaction or single‐term predictor from the most complex models (see Table S3). Model residuals were visually inspected before ANOVA and Tukey's post‐hoc tests were performed. Consequently, the final model formulation for the time series analysis of the landscape diversity was:

$$\begin{array}{c} \mathrm{Sh}\_\mathrm{div}∼f\left(\text{NumDays},3\right)+\text{Temp}\_\text{Regime}+\text{Corridor}\\ +f\left(\text{NumDays},3\right):\text{Temp}\_\text{Regime}+f\left(\text{NumDays},3\right)\\ :\text{Corridor}+\text{ar1}\left(\text{NumDays}\right)+\left(1|\text{Replicate}\right). \end{array}$$

We then tested for differences across the temperature treatments and corridor lengths at the end of the experiment. We performed two‐way ANOVAs using landscape diversity and beta diversity of the last day as dependent variables in two distinct analyses, using temperature regime, the corridor length and the interaction between the two as categorical explanatory variables. To validate the assumptions of the two‐way ANOVA, the residuals of the models were checked for normality homogeneity of variance using Shapiro–Wilk's normality test and Levene's test.

Finally, to visualise if protist community composition (i.e., species identity and abundances) differed between landscapes, we performed principal component analyses (PCA) on the landscape‐level abundances at day 14 (the midpoint) and day 28 (the final day of sampling). Each species' abundance was scaled to mean zero and unit variance prior to analysis to ensure each species were equally weighted. To investigate the effects of temperature regime and corridor length on landscape community composition, we performed permutation ANOVA (PERMANOVA) on the Bray–Curtis dissimilarity matrix constructed upon the scaled species abundances using the ‘adonis2’ function in the ‘vegan’ package. We also performed permutational analysis of dispersions (PERMDISP), to quantify compositional variance (hereafter ‘community dispersion’), using the ‘betadisper’ function in vegan. In combination, PERMANOVA and PERMDISP can provide a measure of across‐and within‐treatment differences, respectively (Anderson et al., 2006; Pu & Jiang, 2015). Because a significant PERMDISP reveals that communities differ in terms of within‐treatment variation, this indicates that the respective PERMANOVA result may be due to high levels of variability between replicates, rather than community composition per se. To disentangle these differences further, we conducted pairwise post‐hoc analyses to investigate which temperature regimes differed from each other.

## 3 RESULTS

### 3.1 Temporal dynamics

**FIGURE 2:**
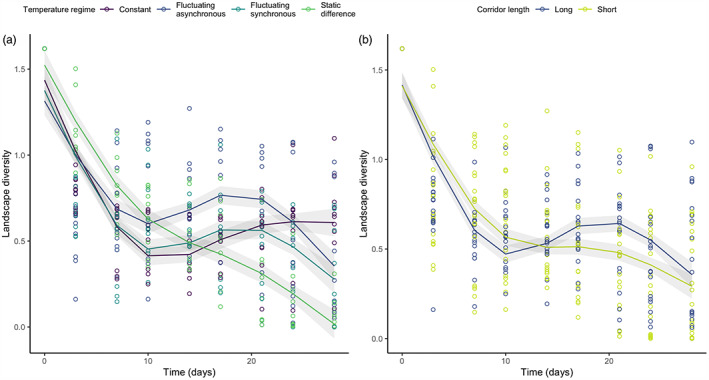
Predicted (lines) and observed (points) values of landscape‐level Shannon diversity index through the duration of the experiment across (a) the four temperature regimes (pooled across corridor lengths) and (b) the two corridor lengths (pooled across temperature regimes).

Time (*χ* ^2^_3,25_ = 202.466, *p* < 0.001), temperature regime (*χ* ^2^_3,25_ = 16.835, *p* = 0.008), the interaction between time and temperature regime (*χ* ^2^_9,25_ = 96.633, *p* < 0.001), and the interaction between time and corridor length (*χ* ^2^_3,25_ = 14.072, *p* = 0.003) had significant effects on landscape diversity (Figure 2; Table 1; Table S4). Thus, across corridor lengths, the temperature regimes differed in how they drove landscape diversity patterns through time (Figure 2a). All experimental landscapes experienced declines in landscape diversity until day 10 when differences between temperature regimes emerged, with diversity continuing to decline in the ‘static difference’ treatment, increasing in the ‘constant’ treatment, or increasing then decreasing in the ‘fluctuating synchronous’ and ‘fluctuating asynchronous’ treatments (Figure 2a; Table 1). Likewise, corridor length significantly altered the trajectory of landscape diversity through time (Figure 2b). Long‐corridor landscapes appeared to exhibit a steeper initial decline in landscape diversity followed by a slight diversity increase around the midpoint of the experiment. Although the ANOVA suggested that across all time points the temperature regimes differed in average landscape diversity (Table 1), such differences did not emerge in the post‐hoc analysis (Table S5). Furthermore, alpha (patch‐level) and gamma (landscape‐level) richness through time have been plotted for each treatment combination to clearly visualise patterns of species loss (Figures S3 and S4).

**TABLE 1:** Anova table of landscape‐level Shannon diversity index analysis through time. Significant effects are in bold

Shannon diversity index ANOVA	Chisq	df	Pr(>*F*)
**ns(NumDays,3)**	**202.466**	**3**	**<2.2 e‐16**
**Temp_Regime**	**16.835**	**3**	**0.008**
Corridor	0.863	1	0.353
**ns(NumDays,3):Temp_Regime**	**96.633**	**9**	**<2.2 e‐16**
**ns(NumDays,3):Corridor**	**14.072**	**3**	**0.003**

### 3.2 Final community diversity

**FIGURE 3:**
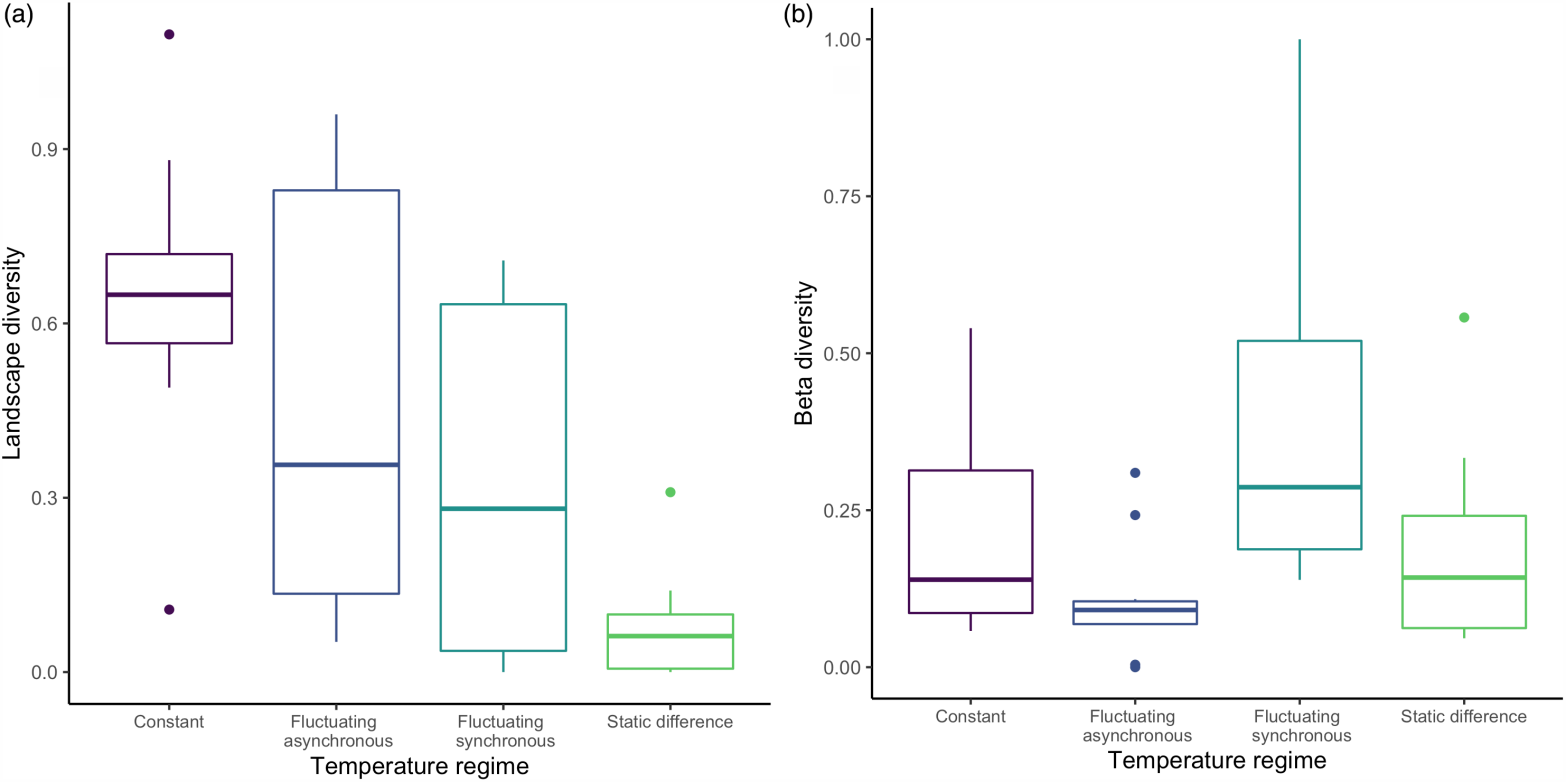
Box plots for the effects of temperature regime on (a) landscape diversity (Shannon diversity index) in each landscape and (b) beta diversity (Bray–Curtis dissimilarity index) between the two patches of each landscape at the end of the experiment (data from long‐ and short‐corridor landscapes pooled together). Outliers are indicated by points with whiskers extending 1.5 times the interquartile range.

At the end of the experiment (day 28), landscape diversity did not differ between the landscapes with long and short corridors (*F*_1,31_ = 0.324, *p* = 0.573) or between the temperature treatments with different corridor lengths (i.e., Corridor:Treatment interaction, *F*_3,31_ = 0.078, *p* = 0.971). However, we found an overall significant effect of temperature regime on landscape diversity (*F*_3,31_ = 6.130, *p* = 0.002). In particular, the static difference landscapes had lower landscape diversity compared with the constant (Bonferroni adjusted *p* < 0.001) and to the fluctuating asynchronous landscapes (Bonferroni adjusted *p* = 0.0345; Figure 3a).

A similar pattern was found for beta diversity, with no effect of corridor length (*F*_1,31_ = 2.738, *p* = 0.108) or corridor length: temperature treatment interaction (*F*_3,31_ = 0.961, *p* = 0.423), whereas temperature treatment had a significant effect on beta diversity (*F*_3,31_ = 3.414, *p* = 0.029). This was driven by the fluctuating synchronous landscapes showing significantly higher values of inter‐patch dissimilarity than the fluctuating asynchronous landscapes (*p* = 0.022, Figure 3b).

### 3.3 Community composition

**FIGURE 4:**
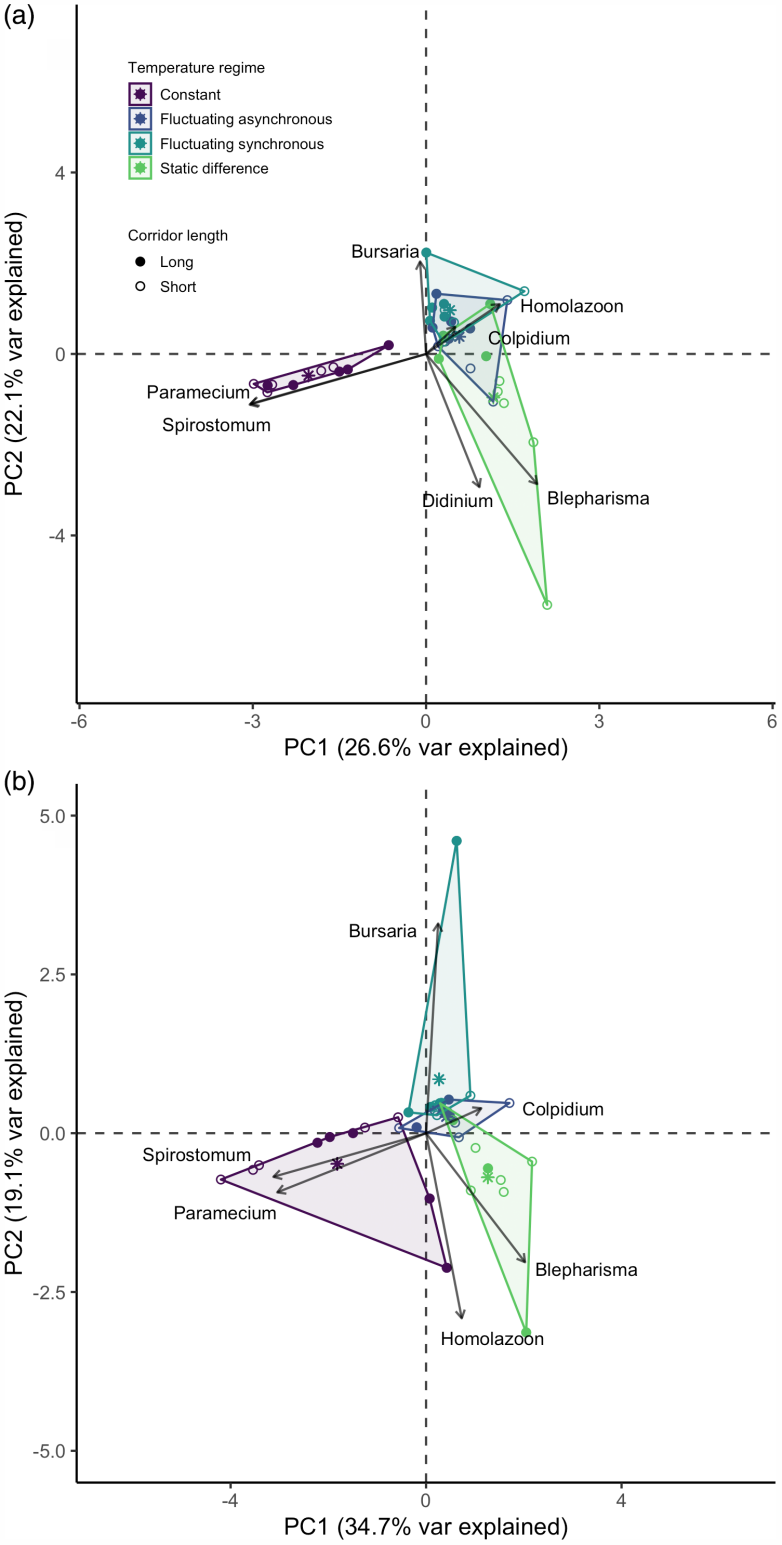
Principal component plots of protist communities sampled in 39 microcosms at (a) 14 days and (b) 28 days. Each point represents a whole landscape community, with colour denoting temperature treatment and fill denoting corridor length. The first two components account for 48.7% (a) and 53.8% (b) of the variation in the data.

Temperature regime had significant effects on landscape‐level community composition at days 14 and 28, and corridor length had significant effects on community composition at day 14 but not at day 28 (PERMANOVA; Figure 4; Table 2). However, temperature regime also significantly affected community dispersion at days 14 and 28 and corridor length significantly affected community dispersion at day 14 but not 28 (PERMDISP; Figure 4, Table 2). A strong difference in community dispersion indicates that any observed community composition differences may result from the differing dispersion (i.e., within‐treatment variation) rather than composition (i.e., the between‐treatment variation). We therefore consider high community dispersion as lower community determinism through time, or that the final community composition is not as predetermined in high dispersion treatments compared with low dispersion treatments. The significant PERMANOVA may be an artefact of high levels of variability between replicates, as indicated by the higher PERMDISP.

**TABLE 2:** Results of the PERMANOVA and dispersion (PERMDISP) analysis of community composition measured using Bray–Curtis dissimilarity after half of the experiment (14 days) and at the end (28 days). Significant effects are in bold

Treatment	PERMANOVA				PERMDISP			
	df	S.sq	*F*‐value	*p*‐value	df	S.sq	*F*‐value	*p*‐value
14 days								
Temperature regime	3	4.9843	12.3227	**0.001**	3	0.17246	2.8499	**0.042**
Corridor length	1	0.6541	4.8512	**0.003**	1	0.13080	12.3	**0.002**
28 days								
Temperature regime	3	5.7264	10.452	**0.001**	3	0.35779	4.8806	**0.007**
Corridor length	1	0.3123	1.710	0.145	1	0.00961	0.9689	0.356

Communities in the constant temperature regime had significantly lower dispersion than communities in the fluctuating asynchronous and static difference temperature regimes at day 14, indicating lower levels of within‐treatment variation (PERMDISP; Figure 4a, Table 2; Table S6). In addition, at day 14 communities in short‐corridor landscapes had significantly higher dispersion than communities in long‐corridor landscapes (PERMDISP; Figure 4a, Table 2; Table S6). Finally, at day 14 community composition of the fluctuating synchronous temperature regime was significantly different from community composition in the constant and static difference temperature regimes (PERMANOVA; Figure 4a, Table 2; Table S6).

At day 28, communities in the fluctuating synchronous temperature regime had significantly lower dispersion than communities in all other temperature regimes, indicating lower within‐treatment variation and consequently more deterministic communities in the fluctuating synchronous treatment (PERMDISP; Figure 4b; Table 2, Table S6). Furthermore, the following communities were compositionally distinct (i.e., significant differences in community composition between treatments) from one another at day 28: constant and fluctuating asynchronous, constant and static difference, fluctuating asynchronous and static difference (PERMANOVA; Figure 4b; Table 2; Table S6).

## 4 DISCUSSION

It is imperative to understand the effects of spatiotemporal variation in temperature to predict future biodiversity change. Here we investigated this by manipulating spatial, synchronous and asynchronous variation in temperature in two‐patch landscapes connected by long or short linear corridors and tracking the responses of a protist community through time. We reveal that thermal variation can significantly alter the dynamics, diversity and composition of a community. Specifically, landscapes with spatial—but not temporal—variation in temperature were characterised by continuous declines in diversity throughout the experiment's duration, resulting in distinct communities with lower diversity than constant or fluctuating asynchronous landscapes. Landscapes subjected to synchronous fluctuation had the lowest levels of within‐treatment variation at the end of the experiment, indicating that these communities were the most deterministic—i.e., displayed more consistent final communities between replicates than the other treatments. Finally, while corridor length did alter the temporal dynamics of diversity and levels of within‐treatment variation, these effects were transient as corridor length had no effect by the end of the experiment.

Supporting previous experimental evidence that temporal thermal variation alters trends of diversity change (Rasconi et al., 2017), we found temperature regime to significantly alter the trajectory of landscape diversity (Figure 2a; Table 1). Contrary to hypothesis 1, constant landscapes had the highest diversity at the end of the experiment, although hypothesis 1's expectations were met in intermediate phases of the experiment. The general trend of diversity decline and species loss followed by differential trajectories depending on the temperature regime can be explained by a combination of the treatment conditions' direct effects on species growth rates and carrying capacities (Table S2) and indirect effects on species interactions (Meester et al., 2015; Trew & Maclean, 2021). Thermal variation has been shown to cause decline of large‐bodied species which are unable to adapt quickly (Rasconi et al., 2017), and higher trophic levels which can be more sensitive to changes in environmental conditions such as warming (Petchey et al., 1999). However, we found the constant landscapes—the only temperature regime to have no thermal variation—to be characterised by early extinctions of many species including *Didinium nasutum* and *Homalozoon vermiculare*, both of which are voracious predators of *Paramecium caudatum* (Baumberg & Hausmann, 2007; Hewett, 1988). Species interactions are fundamental in structuring species responses to environmental change (Daugaard et al., 2019; Jiang & Kulczycki, 2004; Robertson & Hammill, 2021), and indeed these early extinctions relaxed competitive and predation pressures on *Paramecium caudatum* and *Spirostomum teres*. This enabled their abundances to increase, thus increasing the landscape Shannon diversity at the end of the experiment and conflicting with the expectations of hypothesis 1. Similarly, this two species dominance across the replicates culminated in the constant landscape being more deterministic (i.e. lower community dispersion) than the static difference and fluctuating asynchronous regimes at the midpoint of the experiment (Figure 4a,b; Table 2; Table S6). This trend mirrors previous findings of extinctions being followed by an increase in abundance of those that persist, such as fast‐reproducing, small‐bodied species (Petchey et al., 1999; Rasconi et al., 2017).

The differential patterns of diversity change led to fluctuating synchronous landscapes being slightly higher in landscape diversity than the constant treatment (Figure 2) and compositionally distinct from both constant and static difference landscapes at day 14 (Figure 4a; Table 2; Table S6). Such results indicate a non‐linear effect of temperature fluctuation on biological rates (hypothesis 1) as suggested by Jensen's inequality (Jensen, 1906). Jensen's inequality posits that increased variance around an average driver can result in higher or lower average response rates compared with the result predicted by the average driver (Ruel & Ayres, 1999). Here, despite the constant and the fluctuating synchronous landscapes having the same average temperature (i.e. 20°C), the physiological rates of the species may have been comparatively higher in the fluctuating temperature landscape (i.e. positive effect of the ‘sub‐optimal’ conditions induced by temperature variance Martin & Huey, 2008), promoting diversity at the middle phase of the experiment. These differences were driven by increased abundances of *Bursaria truncatella* and *Homalozoon vermiculare* (Figure S2; Figure 4a), in agreement with previous findings that temporal thermal variation also increases predator survival (Hammill & Dart, 2022). This indicates the potential for temporal thermal variation to buffer against species loss, providing evidence for hypothesis 1b at the experiment's midpoint. As increased temperatures can increase the strength of predator–prey interactions (Robertson & Hammill, 2021), the 12‐h 15°C period likely provided a form of temporal refuge, reducing predation pressure and enabling prey abundance to increase before temperature and thus predation pressure increased for the next 12 h.

Fluctuating synchronous landscapes also showed significantly higher beta diversity than fluctuating asynchronous landscapes (Figure 3b). This runs contrary to the processes behind hypotheses 1a and 1c, which predict that landscapes with spatial temperature variation (i.e., static difference and fluctuating asynchronous) would have higher beta diversity due to different‐temperature patches being occupied by distinct communities as a result of species sorting (Chesson & Warner, 1981). Such result might be explained by the combined spatial and temporal temperature variation in the fluctuating asynchronous treatment homogenising the communities by causing extinction of species that were unable to withstand the frequent temperature changes—in this case all of the apex predators (Figure S2)—and dominance of the survivors—in this case, *Blepharisma japonicum* (Figure S2). In addition, it is possible that little dispersal occurred within the fluctuating synchronous landscapes due to both patches being the same temperature (i.e. no gradient of environmental cues to be followed; Laurent et al., 2020), culminating in distinct communities due to a lack of the homogenising effect of dispersal (Mouquet & Loreau, 2003).

While fluctuating synchronous landscapes had higher beta diversity than fluctuating asynchronous landscapes, they also had significantly lower levels of community dispersion than all other temperature regimes, indicating that the synchronous treatment imposed a strong deterministic effect on the final communities (Figure 4b; Table 2; Table S6). This is due to the nature of this temperature regime which meant that, at any one time, both patches in a landscape were the same temperature. As such, if any one species was unable to persist in either of the two temperatures, it would not survive due to a lack of thermal refugia, an important form of spatial refuge in landscapes with thermal variation (Brewitt & Danner, 2014). While neither of the temperatures were sufficiently extreme to directly cause mortality of the prey in our experiment (Table S2), the warmer 12 h may have exacerbated competitive antagonism or predation pressure (Jiang & Kulczycki, 2004; Robertson & Hammill, 2021), causing extinctions. In addition, across all replicates of the fluctuating synchronous treatment only a single predator individual remained (Figure S2), mirroring previous experimental evidence that warming causes extinction of top predators (Petchey et al., 1999), and providing further evidence for temporal thermal fluctuation causing extinction of particular species (Rasconi et al., 2017).

The continual decline of landscape diversity in static‐difference landscapes (Figure 2a) culminated in them having significantly lower diversity than, and being compositionally distinct from, constant and fluctuating asynchronous landscapes (Figures 3a and 4b). We had hypothesised that static‐difference landscapes would have higher diversity than constant landscapes due to niche partitioning caused by species sorting (hypothesis 1a; Chesson & Warner, 1981), and the previously documented coexistence‐promoting effect of thermal variation (Jiang & Morin, 2007). Nevertheless, spatial variation can cause the occupiable niche space in a landscape to decrease if certain patches in the landscape are unfavourable for multiple species. This can even exacerbate the effects of an interaction, for example in an experiment where spatial variation intensified a predator–prey interaction to eventually cause extinction (Salt et al., 2017). As warmer temperatures increase predator–prey interaction strengths (Robertson & Hammill, 2021), it is possible that increased predation pressure led to declines in the warmer patches. Furthermore, as all the prey species in our experiment have higher growth rates at 25°C than at 15°C (Table S2), it is likely that competitive intensity was higher in the warmer patch (as predicted by the metabolic theory of ecology; Brown et al., 2004), leading to population declines of the competitively inferior species and dominance of the competitively superior species. This is illustrated by dominance of *Blepharisma japonicum* in the static‐difference landscapes compared with dominance of *Paramecium caudatum* and *Spirostomum teres* in the constant landscapes (Figure S2), resulting from *Blepharisma japonicum* having a broader thermal tolerance and optimal metabolic performance in the higher temperature patch (Table S2; Matsuoka et al., 1990). With the warmer patch acting as a highly productive source environment, *Blepharisma japonicum* individuals may have gained competitive superiority over the other species (Fox & Morin, 2001), in addition to occasionally being able to predate them (Giese & Smith, 1973). Therefore, the warmer patch increased interaction strengths, ultimately excluding prey species and reducing landscape diversity.

Higher landscape diversity in fluctuating asynchronous landscapes than static‐difference landscapes (Figure 3a) may be explained by the stress of the fluctuating asynchronous temperature regime, with a regular altering of patch temperatures. When coupled with immigration via dispersal, temporal temperature variation has been shown to generate temporary periods of negative population growth and consequently reduced abundances of a competitively superior species, thus permitting persistence of a competitively inferior species (Long et al., 2007). This provides a potential explanation for the patterns seen here, with the temperature fluctuation resulting in predator extinctions (Figure S2) and also preventing dominance of the omnivorous *Blepharisma japonicum*, ultimately permitting higher diversity of prey species via the coexistence‐promotion of spatiotemporal variation (process behind hypothesis 1c, Jiang & Morin, 2007).

Overall, despite our prediction that corridor length would affect diversity responses to the temperature treatments (hypothesis 2), landscape diversity was not shaped by a three‐way interaction between corridor length and temperature regime though time (Table S3). The influence of temperature regime is therefore likely more impactful on species survival patterns than corridor length. In fact, while—across replicates—the same temperature treatments show variability in the trends of some species between long and short corridors (e.g., higher variance in *Blepharisma japonicum* abundance trends in the static‐difference landscapes with long corridors, Figure S2), the overall patterns through time are consistent. This implies that, when coupled with another stressor such as thermal variation, the effects of increased dispersal through corridors can be diminished.

While we also observed a weak overall effect of corridor length on the trajectory of landscape diversity through time (Table 1; Figure 2b), the trends are similar across corridor lengths. We therefore cannot exclude that the apparent difference is merely an artefact of low sample size. However, previous experiments have demonstrated that increasing corridor length reduces net movement of dispersers (Li et al., 2021) and dispersal rate of some prey species (Table S1; Figure S1; Donahue et al., 2003; Laurent et al., 2020). We thus hypothesise that between‐patch movement of prey was minimised in the long‐corridor landscapes. For example, as, in the absence of predator dispersal, prey dispersal directly translates to increased predator abundance (Limberger & Wickham, 2011), in the short‐corridor landscapes of our experiment increased prey dispersal directly translated to increased predator abundance. In particular, the low‐mobility predator *Homalozoon vermiculare* maintained higher abundance in the short‐corridor landscapes, likely due to higher between‐patch movement of the prey providing sustained food availability for these slow‐moving individuals. Consequently, prey species abundances were lower in short corridor landscapes (e.g., lower abundances of *Spirostomum teres*), resulting in lower landscape diversity. The same pattern was exhibited for *Didinium nasutum* which, although not directly affected by the corridor length (due to its high movement capacity, Figure S1), benefited from higher ‘prey supply’ due to higher dispersal rates of most of the prey species. Furthermore, lower prey dispersal rates in the long‐corridor landscapes likely drove the more rapid collapse of the predators, exhibited in the initial steeper decline in landscape diversity. Subsequent relaxation of predation pressure then increased prey densities (Figure S2), driving the increase in diversity at the experiment's midpoint (Figure 2b).

As with any experimental system, freshwater ciliate microcosms are somewhat limited in their ability to represent the full remit of dynamics seen in natural systems. However, our experiment did contain a range of important interaction types that structure any community—predation and competition—as well as a variety of feeding modes—omnivory, bacterivory, and predation. We were also able to incorporate metacommunity dynamics in our system via the addition of dispersal corridors, providing an additional layer of realism. Consequently, while our experimental system does not provide an exact replica of natural systems, it incorporates many of the dynamics that are important for structuring biodiversity responses to environmental change.

While general effects of thermal variation are well studied, there remains limited investigation into the differences between different types of thermal variation in how they shape community dynamics. Our experiment showed that different types of thermal variation can affect whether communities are deterministic or stochastic and how community abundance and composition is supported through time. This key finding suggests that continuing to overlook these effects when investigating the impacts of climatic change may significantly increase the uncertainty in how communities will react to continued anthropogenic change.

## AUTHOR CONTRIBUTIONS

Ellie Wolfe, Francesco Cerini, Christopher F. Clements, and Marc Besson conceived the ideas and designed the experiments. Ellie Wolfe, Francesco Cerini, and Marc Besson performed the experiments. Ellie Wolfe, Francesco Cerini, and Duncan O'Brien performed the statistical analyses. Ellie Wolfe and Francesco Cerini wrote the first draft of the manuscript. All authors contributed substantially to the manuscript revisions. Ellie Wolfe and Francesco Cerini contributed equally to the manuscript.

## CONFLICT OF INTEREST

The authors declare they have no conflicts of interest.

## Supporting information


Data S1

## Data Availability

All code and data are available in the Zenodo record: https://doi.org/10.5281/zenodo.7383209 (Wolfe, Cerini, et al., 2022) and the associated GitHub repository: https://github.com/franzmatches/Fluctuating_temperatures.
