# Supplementary material for: Spatiotemporal thermal variation drives diversity trends in experimental landscapes
Source: J Anim Ecol. 2022 Dec 15;92(2):430–41. doi: 10.1111/1365-2656.13867 (PMC10108128; doi:10.1111/1365-2656.13867)
Supplement: Supplementary file 1 — Data S1 [file JANE-92-430-s001.docx]

**Spatiotemporal thermal variation drives diversity trends in experimental landscapes**

Ellie Wolfe*^1^, Francesco Cerini*^1^, Marc Besson^1,2^, Duncan O’ Brien^1^, Christopher F. Clements^1^

^1^School of Biological Sciences, University of Bristol, Bristol, BS8 1TQ, UK

^2^Sorbonne Université CNRS UMR Biologie des organismes marins, BIOM, F-66650 Banyuls-sur-Mer, France

**Correspondence author:**

Ellie Wolfe

School of Biological Sciences, University of Bristol, Bristol, BS8 1TQ, UK

wolfe.ellie@gmail.com

**Table S1**. Table summarising results for two-sample Wilcoxon or ‘Mann-Whitney’ test (W) comparing the mean (across five replicates) number of individuals dispersed (N_ind) to the adjacent patch using short- and long-corridor landscapes, after two or four hours (for prey and predator species, respectively). To match densities used during set-up of the full experiment, density was 10 individuals/ml of medium in the start patch for prey and 1 individual/ml for predators. Bold p-value indicates a significant difference in the number of dispersed individuals, indicating an effect of the corridor length on the dispersal capacity of the species. There was no effect for predator species, and all the prey species had lower dispersal in long-corridors landscapes except *Paramecium caudatum* (but see figure S4). Data extracted from a dataset which can be found at (https://github.com/franzmatches/Fluctuating_temperatures)

| Species | Trophic  class | Time (hours) | N_ind long | N_ind short | W | p |
| --- | --- | --- | --- | --- | --- | --- |
| *B. japonicum* | Prey | 2 | 11.2 | 38.6 | 0 | **0.007** |
| *C. striatum* |  | 2 | 9.8 | 21.8 | 2 | **0.031** |
| *P. caudatum* |  | 2 | 33.6 | 51.6 | 12 | 1 |
| *S. teres* |  | 2 | 14.4 | 34.6 | 2 | **0.036** |
| *D. nasutum* | Predator | 4 | 5 | 5.8 | 10.5 | 0.750 |
| *H. vermiculare* |  | 4 | 0 | 0 | Na | Na |

**Table S2.** Table summarising mean ± SE for growth rates (r)and carrying capacities (k) of the prey species used in our experiment. The experiments were conducted at each of the three different temperatures used in the full experiment (15^o^C, 20^o^C and 25^o^C) and were replicated four times for each species. Abundances were sampled every weekday until carrying capacity appeared to have been reached (i.e., consistent abundances for >3 sampling days). Data extracted from a dataset which can be found at (https://github.com/ExperimentalConservation)

|  | r | | | k | | |
| --- | --- | --- | --- | --- | --- | --- |
| Species | 15^o^C | 20^o^C | 25^o^C | 15^o^C | 20^o^C | 25^o^C |
| *B. japonicum* | 0.018±0.003 | 0.023±0.001 | 0.036±0.002 | 0.008±0.001 | 0.193±0.013 | 0.119±0.009 |
| *C. striatum* | 0.040±0.002 | 0.062±0.002 | 0.088±0.010 | 0.541±0.051 | 0.873±0.059 | 0.654±0.076 |
| *P. caudatum* | 0.027±0.001 | 0.034±0.002 | 0.049±0.004 | 0.044±0.005 | 0.154±0.034 | 0.118±0.023 |
| *S. teres* | 0.027±0.005 | 0.036±0.003 | 0.039±0.004 | 0.005±0.001 | 0.008±0.001 | 0.005±0.001 |

**Table S3.** Model comparison through AICc examining the influence of time (NumDays), temperature regime (constant/fluctuating synchronous/fluctuating asynchronous/static difference) and corridor length (long/short) on the landscape-level Shannon diversity index. The ∆AICc column reports the difference in AICc value for each model relative to the simplest, with the final selected model indicated by a *. The model description column summarises the rationale behind every model predictor

| Model formula | df | AICc | ∆AICc | Model description |
| --- | --- | --- | --- | --- |
| *Sh_div~ f(NumDays,3) + Temp_Regime +*  *Corridor + f(NumDays,3):Temp_Regime + f(NumDays,3):Corridor + ar1(NumDays + 0 \| Replicate) + (1\|Replicate) ** | 24 | 49.2 * | 0.00 * | Model investigating   1. how the overall landscape Shannon diversity changes nonlinearly through time *(f(NumDays,3))* 2. how the intercepts of the different temperature regimes (pooling long and short corridor data) change compared to the constant temperature *(Temp_Regime)* 3. how the regression intercepts of short corridor landscapes (pooling all the temperature regimes) differ from the long corridor landscapes *(Corridor)* 4. how the nonlinear trends of landscape Shannon diversity of the different temperature regimes through time (pooling long and short corridor data) differ from the constant regime trends *(f(NumDays,3): Temp_Regime)* 5. how the nonlinear trends of landscape Shannon diversity of short corridor landscapes though time (pooling all the temperature regime data) differ from the long-corridor regime trends *(f(NumDays,3): Corridor)* |
| *Sh_div~ f(NumDays,3) +Temp_Regime + Corridor + f(NumDays,3):Temp_Regime + f(NumDays,3):Corridor + Temp_Regime:Corridor + ar1(NumDays + 0 \| Replicate) + (1\|Replicate)* | 27 | 55.4 | 6.25 | Model investigating   1. how the overall landscape Shannon diversity changes nonlinearly through time *(f(NumDays,3))* 2. how the intercepts of the different temperature regimes (pooling long and short corridor data) change compared to the constant temperature *(Temp_Regime)* 3. how the regression intercepts of short corridor landscape (pooling all the temperature regimes) differ from the long corridor *(Corridor)* 4. how the nonlinear trends of landscape Shannon diversity of the different temperature regimes through time (pooling long and short corridor data) are different from the constant regime trend *(f(NumDays,3): Temp_Regime)* 5. how the nonlinear trends of landscape Shannon diversity of short corridor landscapes though time (pooling all the temperature regimes data) are different from the long-corridor regime trend *(f(NumDays,3): Corridor)* 6. how the intercept of all the combination of corridors lengths and temperature regimes differ from the long-corridor constant-temperature landscape (*Temp_Regime:Corridor)* |
| *Sh_div~ f(NumDays,3) +Temp_Regime + Corridor + f(NumDays,3):Temp_Regime + f(NumDays,3):Corridor + Temp_Regime:Corridor + f(NumDays,3):Temp_Regime:Corridor + ar1(NumDays + 0 \| Replicate) + (1\|Replicate)* | 36 | 61.1 | 11.89 | Model investigating   1. how the overall landscape Shannon diversity changes nonlinearly through time *(f(NumDays,3))* 2. if the regression intercepts of the different temperature regimes (pooling long and short corridor data) differ compared to the constant temperature which is used as baseline *(Temp_Regime)* 3. how the regression intercepts of short corridor landscape (pooling all the temperature regimes) differ from the long corridor which is used as baseline *(Corridor)* 4. how the nonlinear trends of landscape Shannon diversity of the different temperature regimes through time (pooling long and short corridors data) differ from the constant regime trend *(f(NumDays,3): Temp_Regime)* 5. how the nonlinear trends of landscape Shannon diversity of short-corridor landscapes though time (pooling all the temperature regimes data) differ from the long-corridor regime trend *(f(NumDays,3): Corridor)* 6. how the regression intercepts of each combination of corridor lengths and temperature regimes differ from the constant-temperature long-corridor landscape (*Temp_Regime: Corridor)* 7. how the nonlinear trends of landscape Shannon diversity though time of all the combinations of corridors lengths and temperature regimes differ from the constant- temperature long-corridor landscape (*f(NumDays,3): Temp_Regime: Corridor)* |

*f(NumDays,3):* natural cubic spline function with three knots for the *NumDays* variable

*Sh_div:* the Shannon diversity index

*NumDays:* the time variable (with days as units)

*Temp_Regime*: the temperature treatment used in the experiment (categorical variable with four levels)

*Corridor:* whether a long or short corridor separated the two patches (categorical variable with two levels).

*ar1(NumDays + 0 | Replicate):* a first-order autocorrelation term controlling for excess serial dependency in the model residuals. Individual terms as estimated for each random effect level.

*(1|Replicate*): random effect term for each replicate level.

Interactions between variables are indicated by a colon.

**Table S4.** *Parameter estimates for landscape diversity according to temperature regime and corridor length through time. Random effect sizes are presented along with the model deviance and degrees of freedom.*

| **Random effects** | **Variance** | **Std.Dev** | **Corr** |
| --- | --- | --- | --- |
| Replicate | 2.01e-13 | 4.483e-07 |  |
| ar(1) | 1.55e-02 | 1.245e-01 | -0.19 |
|  | Number of observations: 351 | Number of groups: 5 | Deviance: -2.15 |
| **Fixed effects** | **Estimate** | **Confidence interval** | **P value** |
| Intercept | 1.441 | 1.353 – 1.529 | <2e-16 *** |
| F(NumDays, 3)1 | -0.285 | -0.414 – -0.156 | 0.028 * |
| F(NumDays, 3)2 | -1.949 | -2.167 – -1.730 | <2e-16*** |
| F(NumDays, 3)3 | -0.026 | -0.120 – 0.068 | 0.782 |
| Temp_regime (fluctuating asynchro) | -0.121 | -0.208 – -0.034 | 0.163 |
| Temp_regime (fluctuating synchro) | -0.060 | -0.147 – 0.026 | 0.487 |
| Temp_regime (static difference) | 0.088 | -0.001 – 0.178 | 0.324 |
| Corridor (short) | -0.011 | -0.074 – 0.051 | 0.857 |
| F(NumDays, 3)1: Temp_regime (fluctuating asynchro) | 0.403 | 0.266 – 0.541 | 0.003** |
| F(NumDays, 3)2: Temp_regime (fluctuating asynchro) | 0.336 | 0.116 – 0.555 | 0.126 |
| F(NumDays, 3)3: Temp_regime (fluctuating asynchro) | -0.303 | -0.522 – -0.084 | 0.003** |
| F(NumDays, 3)1: Temp_regime (fluctuating synchro) | 0.164 | 0.026 – 0.305 | 0.235 |
| F(NumDays, 3)2: Temp_regime (fluctuating synchro) | -0.040 | -0.260 – 0.179 | 0.855 |
| F(NumDays, 3)3: Temp_regime (fluctuating synchro) | -0.320 | -0.421 – -0.220 | 0.001** |
| F(NumDays, 3)1: Temp_regime (static difference) | -0.332 | -0.474 – -0.190 | 0.019* |
| F(NumDays, 3)2: Temp_regime (static difference) | -0.311 | -0.537 – -0.085 | 0.169 |
| F(NumDays, 3)3: Temp_regime (static difference) | -0.826 | -0.929 – -0.722 | 1.62e-15*** |
| F(NumDays, 3)1: Corridor (short) | -0.302 | -0.400 – -0.203 | 0.002** |
| F(NumDays, 3)2: Corridor (short) | 0.090 | -0.068 – 0.247 | 0.570 |
| F(NumDays, 3)3: Corridor (short) | -0.165 | -0.234 – -0.093 | 0.022* |
| * p < 0.05 ** p <0.01 ***p<0.001 | |  |  |

| Shannon diversity Index ANOVA | Sum Sq | Df | F values | Pr(>F) |
| --- | --- | --- | --- | --- |
| Temp_Regime | 0.840 | 3 | 1.479 | 0.22 |
| Residuals | 65.78 | 347 |  |  |

**Table S5.** Anova table of the overall effect of temperature regime on Shannon diversity

Index across all time points.

**Table S6.** Between-treatment comparisons of community composition (PERMANOVA) and dispersion (PERMDISP) measured using Bray-Curtis dissimilarity. P-values were computed based on 999 permutations and significance levels were subjected to Bonferonni correction.

| Comparison | PERMANOVA | PERMDISP |
| --- | --- | --- |
| Day 14 | p | p |
| Constant * FA | **0.006** | **0.004** |
| Constant * FS | **0.006** | 0.354 |
| Constant * SD | **0.006** | **0.023** |
| FA * FS | 1 | 0.166 |
| FA * SD | 0.078 | 0.580 |
| FS * SD | **0.018** | 0.405 |
| Short * Long corridors | **0.003** | **0.002** |
| Day 28 |  |  |
| Constant * FA | **0.006** | 0.395 |
| Constant * FS | **0.006** | **0.003** |
| Constant * SD | **0.006** | 0.960 |
| FA * FS | **0.006** | **0.007** |
| FA * SD | **0.006** | 0.605 |
| FS * SD | **0.012** | **0.022** |
| Short * Long corridors | 0.145 | 0.356 |

**

**

**Fig. S1** Frequency histograms of the value of the difference between mean (among 5 replicates) individuals dispersed in long vs short corridor landscapes, resulting from a 1000-permutations bootstrapping of the data derived from experiments. The blue dotted line indicates the observed data from our experiments. The red lines mark the 95% interval.

For three out of four prey species (*B.japonicum, C. striatum* and *S. teres*), a value of 0 in the difference between individuals dispersed in long and short corridors falls out of the histogram’s confidence interval. We can interpret this as a strong effect of the corridor length on the dispersal rate (i.e. the same mean value of individuals dispersed in long and short corridor never occurred in 1000 bootstrapping simulations of the observed data). As for *P.caudatum*, although a zero difference is somehow likely to happen, we can see that a large proportion of the distribution falls after the 0, and we can interpret this as a partial effect of the corridor length on its dispersal in the majority of simulated cases. For *D. nasutum* there is a clear lack of effect. The predator *H. vermiculare* showed zero dispersal in all replicates in both long and short corridors landscapes, therefore it is not shown.


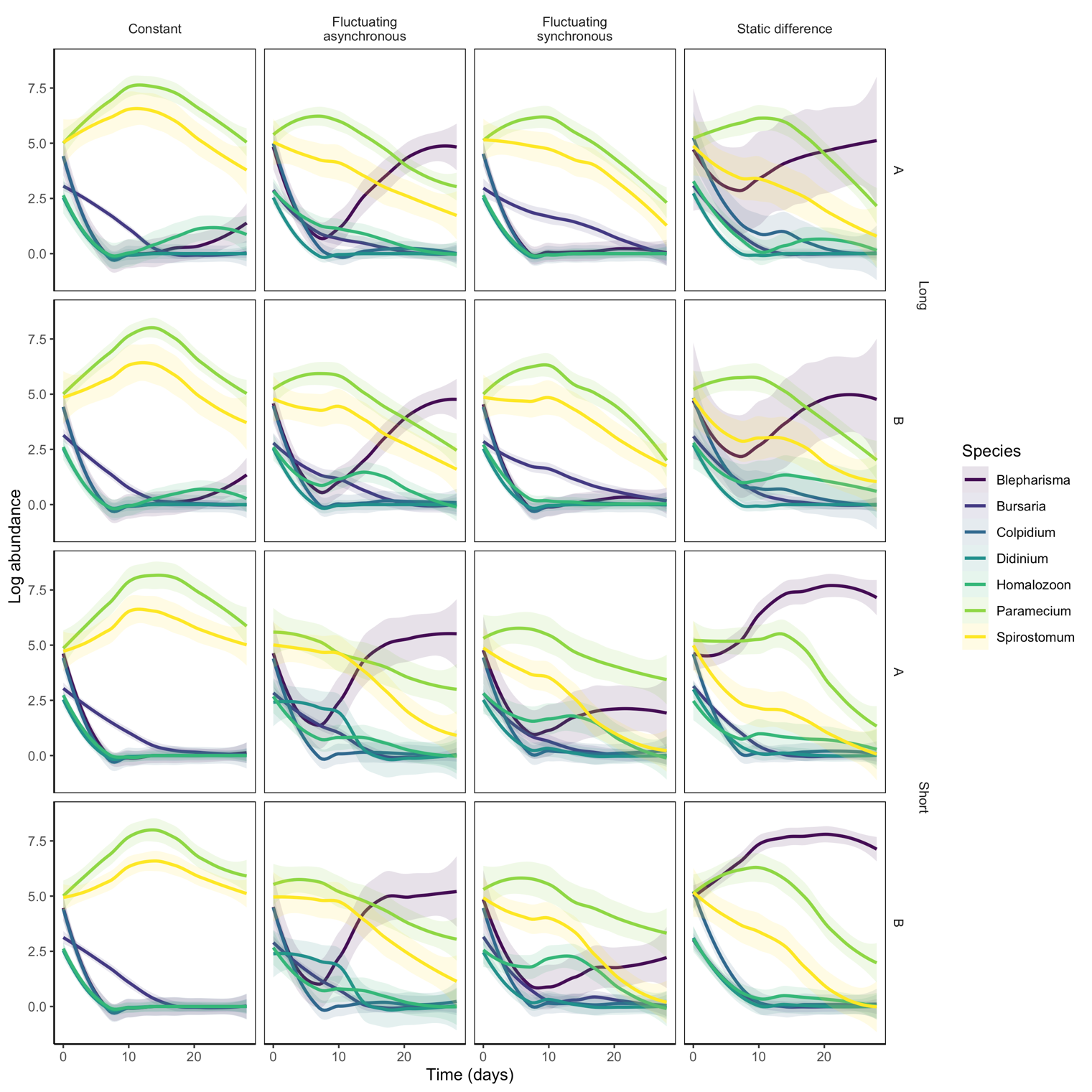


**Fig. S2** Patch-level abundances of each species through time. Columns are temperature regimes and rows are patch-level corridor lengths. Labels A and B refer to individual patches within the landscape


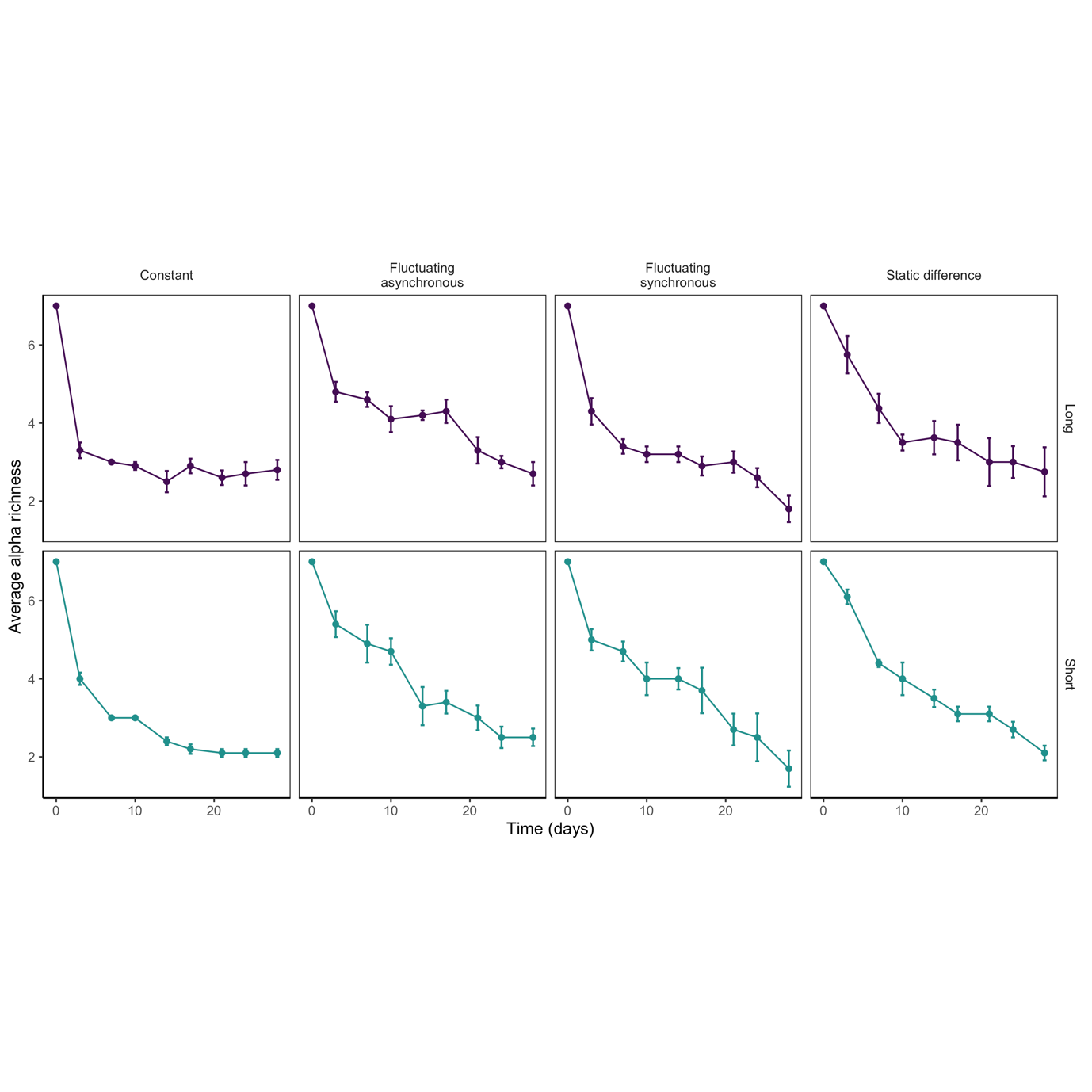


**Fig. S3** Average alpha richness (patch-level richness averaged across both patches in a landscape) through time. Error bars denote mean landscape richness across replicates with standard errors.


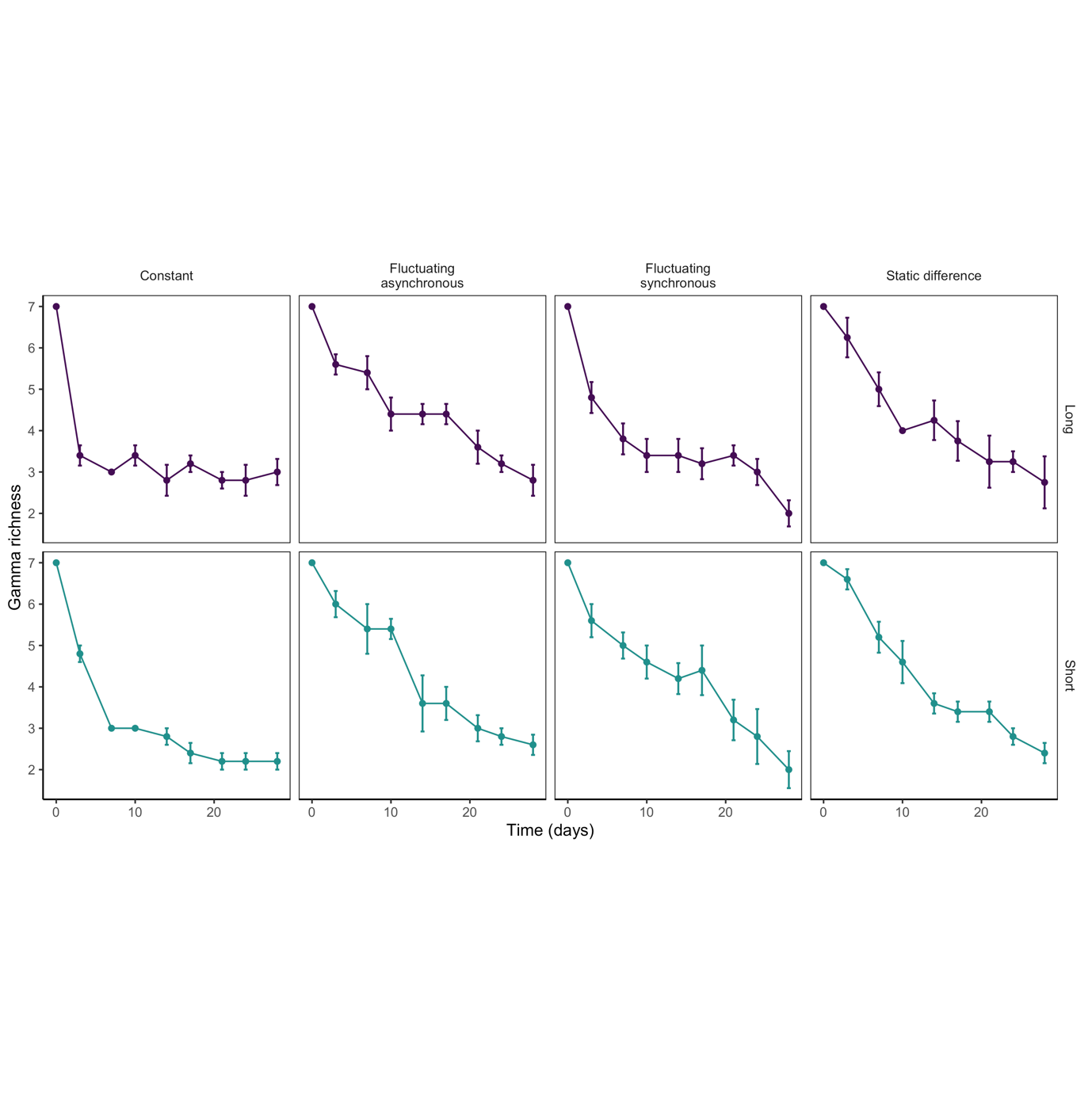
**Fig. S4** Gamma (landscape-level, i.e., the total number of species in a whole landscape) richness through time. Error bars denote mean landscape richness across replicates with standard errors.
